# Bis(2-benzamido­benzimidazolato-κ^2^ *N* ^1^,*O*)(*N*,*N*-dimethyl­formamide-κ*O*)copper(II)

**DOI:** 10.1107/S1600536808016383

**Published:** 2008-06-07

**Authors:** Elhadj Ibrahima Thiam, Farba Tamboura, Mohamed Gaye, Abdou Salam Sall, Aliou Hamady Barry

**Affiliations:** a Département de Chimie, Faculté des Sciences et Techniques, Université Cheikh Anta Diop, Dakar, Senegal; b Département de Chimie, Faculté des Sciences, Université de Nouakchott, Nouakchott, Mauritania

## Abstract

In the title compound, [Cu(C_14_H_10_N_3_O)_2_(C_3_H_7_NO)], the Cu^II^ atom is five-coordinated by two *N*,*O*-bidentate 2-benzamido­benzimidazolate anions and one *O*-coordinated dimethyl­formamide (DMF) mol­ecule, resulting in a distorted square-based pyramidal CuN_2_O_3_ geometry for the metal atom, with the DMF O atom at the apical site. In the crystal structure, inter­molecular N—H⋯N hydrogen bonds result in chains of mol­ecules propagating along [100].

## Related literature

For background on distorted copper coordination geometries, see: Hathaway (1973 ▶).
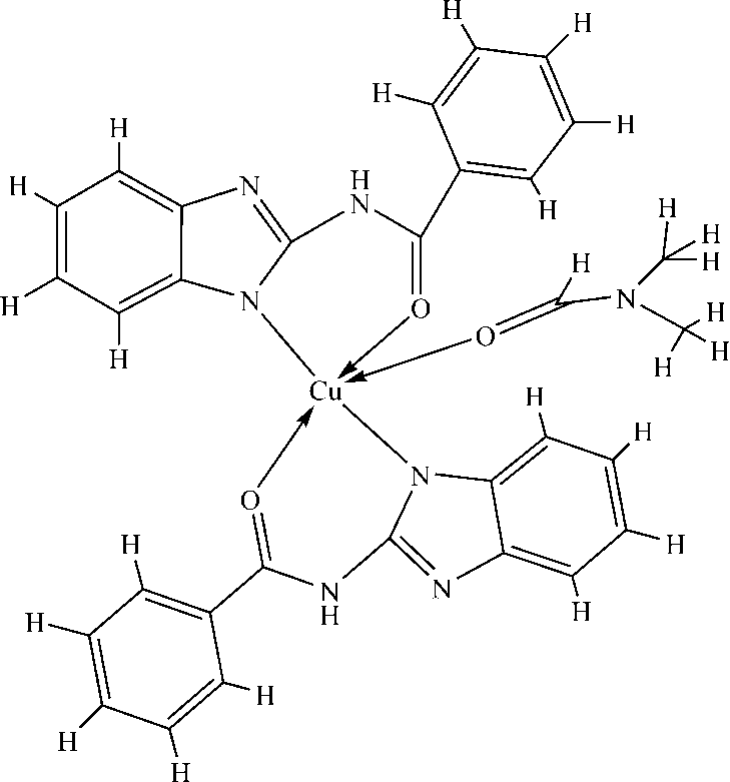


## Experimental

### 

#### Crystal data

[Cu(C_14_H_10_N_3_O)_2_(C_3_H_7_NO)]
*M* *_r_* = 609.14
Monoclinic, 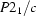

*a* = 9.8798 (2) Å
*b* = 23.3089 (9) Å
*c* = 12.0765 (4) Å
β = 106.427 (2)°
*V* = 2667.54 (15) Å^3^
*Z* = 4
Mo *K*α radiation
μ = 0.87 mm^−1^
*T* = 173 (2) K
0.15 × 0.10 × 0.07 mm

#### Data collection

Nonius KappaCCD diffractometer
Absorption correction: none
22936 measured reflections
7739 independent reflections
5179 reflections with *I* > 2σ(*I*)
*R*_int_ = 0.057

#### Refinement

*R*[*F* ^2^ > 2σ(*F* ^2^)] = 0.051
*wR*(*F* ^2^) = 0.176
*S* = 1.10
7739 reflections
379 parameters
H-atom parameters constrained
Δρ_max_ = 0.79 e Å^−3^
Δρ_min_ = −0.91 e Å^−3^

### 

Data collection: *COLLECT* (Nonius, 1998 ▶); cell refinement: *DENZO* (Otwinowski & Minor, 1997 ▶); data reduction: *DENZO*; program(s) used to solve structure: *SHELXS97* (Sheldrick, 2008 ▶); program(s) used to refine structure: *SHELXL97* (Sheldrick, 2008 ▶); molecular graphics: *PLATON* (Spek, 2003 ▶); software used to prepare material for publication: *SHELXL97*.

## Supplementary Material

Crystal structure: contains datablocks I, global. DOI: 10.1107/S1600536808016383/hb2739sup1.cif

Structure factors: contains datablocks I. DOI: 10.1107/S1600536808016383/hb2739Isup2.hkl

Additional supplementary materials:  crystallographic information; 3D view; checkCIF report

## Figures and Tables

**Table 1:** Selected bond lengths (Å)

Cu1—O2	1.918 (2)
Cu1—O1	1.944 (2)
Cu1—N5	1.945 (2)
Cu1—N2	1.956 (2)
Cu1—O3	2.855 (3)

**Table 2:** Hydrogen-bond geometry (Å, °)

*D*—H⋯*A*	*D*—H	H⋯*A*	*D*⋯*A*	*D*—H⋯*A*
N1—H1N⋯N6^i^	0.88	2.05	2.847 (3)	151
N4—H4N⋯N3^ii^	0.88	2.26	3.019 (3)	144

Symmetry codes: (i) 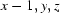; (ii) 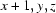.

**Fig. 1.**
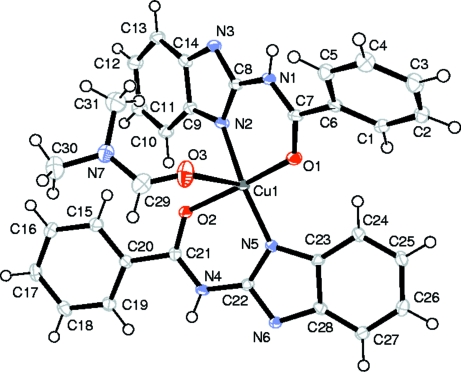
The molecular structure of (I) with displacement ellipsoids for the non-hydrogen atoms plotted at the 50% probability level.

**Fig. 2.**
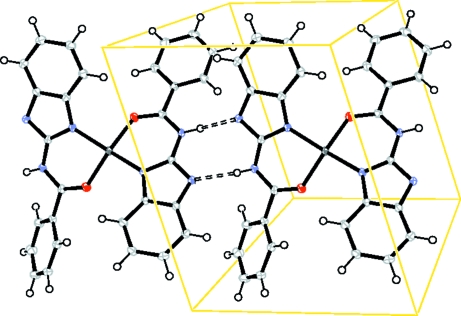
Part of a [100] chain of molecules in the crystal of (I) with hydrogen bonds indicated by double-dashed lines.

## supplementary crystallographic information

### Comment

The title compound, (I), consists of a mononuclear Cu(II) complex with one neutral molecule in the asymmetric unit (Fig. 1). The Cu(II) atom is surrounded by five atoms (Table 1): two molecules of the ligand are linked to the metal atom by one nitrogen atom and one oxygen atom for each moiety and one oxygen atom of a dimethylformamide solvent molecule complete the coordination sphere around the Cu(II) ion. The geometry around the Cu(II) ion is a very distorted square pyramid, with a long apical Cu—O3 bond of 2.8545 (28) Å, which is in the range of "semi-coordination" (2.22–2.89 ° A) for H_2_O (Hathaway, 1973). The dihedral angle between the fused ring system and the phenyl ring in the C1 molecule is 47.83 (13)°; the corresponding angle in the C15 molecule is 4.94 (13)°.

In the crystal, the molecules interact via N—H···N hydrogen bonds (Table 2) to result in chains propagating in [100] (Fig. 2).

### Experimental

A solution of 0.0852 g (0.5 mmol) of copper chloride dihydrate in 10 ml of methanol was added dropwise to a solution of 0.2173 g (0.5 mmol) of 1,2-bis(*N*-benzoylthioureayl)benzene in 10 ml of methanol. A green precipitate appeared immediately. The resulting suspension was stirred at room temperature for 10 min. The compound was filtered and washed with 2 × 10 ml of methanol and dried under vacuum. Recrystallization from dimethylformamide gave 0.1480 g (48.66°) of (I) in the form of yellow prisms. M.p. 493–495 K (uncorrected). IR (cm^-1^, KBr): 3210, 1673, 1596, 1470, 1319, 1262, 1149, 857, 688. Analysis calculated for C_31_H_27_N_7_O_3_Cu: C 61.12, H 4.47, N 16.10%; found: C 61.08, H 4.49, N 16.02%.

### Refinement

All H atoms were placed geometrically (C—H = 0.95–0.98 Å, N—H = 0.88 Å) and refined as riding with *U*_iso_(H) = 1.2*U*_eq_(carrier) or 1.5U_eq_(methyl C).

### Crystal data

**Table:**

[Cu(C_14_H_10_N_3_O)_2_(C_3_H_7_NO)]	*F*_000_ = 1260
*M**_r_* = 609.14	*D*_x_ = 1.517 Mg m^−^^3^
Monoclinic, *P*2_1_/*c*	Mo *K*α radiation λ = 0.71073 Å
Hall symbol: -P 2ybc	Cell parameters from 19475 reflections
*a* = 9.8798 (2) Å	θ = 1.0–30.0º
*b* = 23.3089 (9) Å	µ = 0.87 mm^−^^1^
*c* = 12.0765 (4) Å	*T* = 173 (2) K
β = 106.427 (2)º	Prism, yellow
*V* = 2667.54 (15) Å^3^	0.15 × 0.10 × 0.07 mm
*Z* = 4	

### Data collection

**Table:**

Nonius KappaCCD diffractometer	5179 reflections with *I* > 2σ(*I*)
Radiation source: fine-focus sealed tube	*R*_int_ = 0.057
Monochromator: graphite	θ_max_ = 30.1º
*T* = 173(2) K	θ_min_ = 1.8º
ω and φ scans	*h* = −10→13
Absorption correction: none	*k* = −30→32
22936 measured reflections	*l* = −17→14
7739 independent reflections	

### Refinement

**Table:**

Refinement on *F*^2^	Secondary atom site location: difference Fourier map
Least-squares matrix: full	Hydrogen site location: inferred from neighbouring sites
*R*[*F*^2^ > 2σ(*F*^2^)] = 0.051	H-atom parameters constrained
*wR*(*F*^2^) = 0.177	*w* = 1/[σ^2^(*F*_o_^2^) + (0.0937*P*)^2^ + 0.2242*P*] where *P* = (*F*_o_^2^ + 2*F*_c_^2^)/3
*S* = 1.10	(Δ/σ)_max_ = 0.010
7739 reflections	Δρ_max_ = 0.79 e Å^−^^3^
379 parameters	Δρ_min_ = −0.91 e Å^−^^3^
Primary atom site location: structure-invariant direct methods	Extinction correction: none

### Special details

**Table:**

Geometry. All e.s.d.'s (except the e.s.d. in the dihedral angle between two l.s. planes) are estimated using the full covariance matrix. The cell e.s.d.'s are taken into account individually in the estimation of e.s.d.'s in distances, angles and torsion angles; correlations between e.s.d.'s in cell parameters are only used when they are defined by crystal symmetry. An approximate (isotropic) treatment of cell e.s.d.'s is used for estimating e.s.d.'s involving l.s. planes.
Refinement. Refinement of *F*^2^ against ALL reflections. The weighted *R*-factor *wR* and goodness of fit *S* are based on *F*^2^, conventional *R*-factors *R* are based on *F*, with *F* set to zero for negative *F*^2^. The threshold expression of *F*^2^ > σ(*F*^2^) is used only for calculating *R*-factors(gt) *etc*. and is not relevant to the choice of reflections for refinement. *R*-factors based on *F*^2^ are statistically about twice as large as those based on *F*, and *R*- factors based on ALL data will be even larger.

### Fractional atomic coordinates and isotropic or equivalent isotropic displacement parameters (Å^2^)

**Table:**

	*x*	*y*	*z*	*U*_iso_*/*U*_eq_	
Cu1	0.77484 (3)	0.102988 (16)	0.57415 (3)	0.01523 (12)	
O1	0.6207 (2)	0.08799 (10)	0.43630 (17)	0.0206 (5)	
O2	0.91496 (19)	0.14195 (10)	0.69308 (17)	0.0181 (5)	
O3	0.6892 (3)	0.22000 (12)	0.5355 (2)	0.0363 (6)	
N1	0.4257 (2)	0.10498 (11)	0.5051 (2)	0.0161 (5)	
H1N	0.3343	0.1116	0.4861	0.019*	
N2	0.6414 (2)	0.09987 (11)	0.6666 (2)	0.0154 (5)	
N3	0.4298 (2)	0.10461 (11)	0.7000 (2)	0.0162 (5)	
N4	1.1195 (2)	0.13381 (12)	0.63379 (19)	0.0150 (5)	
H4N	1.2098	0.1423	0.6492	0.018*	
N5	0.9199 (2)	0.08316 (11)	0.5005 (2)	0.0141 (5)	
N6	1.1322 (2)	0.08448 (11)	0.4679 (2)	0.0146 (5)	
N7	0.7081 (3)	0.27521 (13)	0.6965 (2)	0.0235 (6)	
C1	0.4175 (3)	0.05701 (15)	0.2221 (3)	0.0202 (6)	
H1	0.4952	0.0313	0.2434	0.024*	
C2	0.3263 (3)	0.05531 (16)	0.1114 (3)	0.0238 (7)	
H2	0.3383	0.0269	0.0586	0.029*	
C3	0.2177 (3)	0.09480 (16)	0.0773 (3)	0.0253 (7)	
H3	0.1580	0.0947	0.0003	0.030*	
C4	0.1968 (3)	0.13468 (15)	0.1570 (3)	0.0238 (7)	
H4	0.1228	0.1620	0.1337	0.029*	
C5	0.2822 (3)	0.13498 (14)	0.2692 (3)	0.0199 (6)	
H5	0.2642	0.1612	0.3235	0.024*	
C6	0.3954 (3)	0.09662 (14)	0.3029 (2)	0.0174 (6)	
C7	0.4893 (3)	0.09693 (13)	0.4238 (3)	0.0169 (6)	
C8	0.5014 (3)	0.10310 (13)	0.6188 (2)	0.0152 (6)	
C9	0.6610 (3)	0.09950 (13)	0.7858 (2)	0.0163 (6)	
C10	0.7839 (3)	0.09685 (14)	0.8780 (3)	0.0186 (6)	
H10	0.8742	0.0958	0.8647	0.022*	
C11	0.7707 (3)	0.09584 (15)	0.9890 (3)	0.0215 (7)	
H11	0.8532	0.0938	1.0527	0.026*	
C12	0.6375 (3)	0.09771 (15)	1.0095 (3)	0.0221 (7)	
H12	0.6318	0.0967	1.0867	0.026*	
C13	0.5140 (3)	0.10102 (14)	0.9190 (3)	0.0196 (6)	
H13	0.4238	0.1026	0.9325	0.023*	
C14	0.5289 (3)	0.10195 (13)	0.8074 (2)	0.0172 (6)	
C15	1.0370 (3)	0.21264 (15)	0.8703 (3)	0.0196 (6)	
H15	0.9393	0.2039	0.8530	0.024*	
C16	1.0996 (3)	0.24783 (15)	0.9635 (3)	0.0211 (7)	
H16	1.0440	0.2642	1.0080	0.025*	
C17	1.2437 (3)	0.25913 (14)	0.9917 (3)	0.0215 (6)	
H17	1.2873	0.2818	1.0575	0.026*	
C18	1.3231 (3)	0.23727 (14)	0.9238 (3)	0.0210 (6)	
H18	1.4213	0.2453	0.9426	0.025*	
C19	1.2601 (3)	0.20368 (14)	0.8286 (2)	0.0178 (6)	
H19	1.3150	0.1899	0.7810	0.021*	
C20	1.1171 (3)	0.18985 (13)	0.8016 (2)	0.0150 (6)	
C21	1.0449 (3)	0.15228 (13)	0.7022 (2)	0.0143 (6)	
C22	1.0554 (3)	0.10161 (13)	0.5393 (2)	0.0133 (5)	
C23	0.9131 (3)	0.05093 (13)	0.4014 (2)	0.0139 (6)	
C24	0.8023 (3)	0.02016 (14)	0.3286 (2)	0.0177 (6)	
H24	0.7131	0.0180	0.3437	0.021*	
C25	0.8257 (3)	−0.00729 (14)	0.2333 (2)	0.0181 (6)	
H25	0.7513	−0.0281	0.1818	0.022*	
C26	0.9578 (3)	−0.00452 (14)	0.2126 (2)	0.0191 (6)	
H26	0.9704	−0.0228	0.1460	0.023*	
C27	1.0707 (3)	0.02399 (14)	0.2862 (2)	0.0170 (6)	
H27	1.1610	0.0246	0.2729	0.020*	
C28	1.0456 (3)	0.05168 (13)	0.3806 (2)	0.0148 (6)	
C29	0.7481 (4)	0.25614 (16)	0.6062 (3)	0.0286 (8)	
H29	0.8317	0.2725	0.5958	0.034*	
C30	0.7822 (4)	0.31968 (18)	0.7732 (3)	0.0342 (8)	
H30A	0.7344	0.3270	0.8327	0.051*	
H30B	0.7830	0.3549	0.7291	0.051*	
H30C	0.8794	0.3073	0.8098	0.051*	
C31	0.5809 (4)	0.25256 (18)	0.7197 (3)	0.0361 (9)	
H31A	0.5680	0.2709	0.7890	0.054*	
H31B	0.5905	0.2110	0.7318	0.054*	
H31C	0.4989	0.2606	0.6537	0.054*	

### Atomic displacement parameters (Å^2^)

**Table:**

	*U*^11^	*U*^22^	*U*^33^	*U*^12^	*U*^13^	*U*^23^
Cu1	0.00960 (17)	0.0223 (2)	0.01411 (18)	−0.00114 (14)	0.00387 (12)	−0.00258 (15)
O1	0.0123 (9)	0.0329 (14)	0.0159 (10)	−0.0003 (9)	0.0030 (7)	−0.0047 (9)
O2	0.0118 (9)	0.0254 (13)	0.0177 (10)	−0.0017 (8)	0.0049 (7)	−0.0069 (9)
O3	0.0461 (15)	0.0335 (16)	0.0240 (12)	0.0045 (12)	0.0010 (11)	−0.0073 (11)
N1	0.0095 (10)	0.0239 (15)	0.0144 (11)	−0.0015 (10)	0.0028 (8)	−0.0032 (10)
N2	0.0103 (10)	0.0214 (14)	0.0145 (11)	−0.0016 (9)	0.0035 (8)	0.0006 (10)
N3	0.0125 (10)	0.0237 (14)	0.0132 (11)	−0.0018 (10)	0.0047 (9)	−0.0012 (10)
N4	0.0099 (10)	0.0215 (14)	0.0138 (11)	−0.0033 (10)	0.0037 (8)	−0.0010 (10)
N5	0.0103 (10)	0.0179 (13)	0.0136 (11)	−0.0013 (9)	0.0023 (8)	−0.0021 (10)
N6	0.0114 (10)	0.0190 (13)	0.0138 (11)	−0.0018 (10)	0.0044 (8)	−0.0025 (10)
N7	0.0246 (13)	0.0234 (15)	0.0213 (13)	0.0027 (11)	0.0047 (10)	−0.0019 (11)
C1	0.0154 (13)	0.0276 (18)	0.0188 (14)	−0.0016 (12)	0.0068 (11)	−0.0036 (13)
C2	0.0219 (15)	0.035 (2)	0.0152 (14)	−0.0058 (14)	0.0063 (11)	−0.0056 (14)
C3	0.0262 (16)	0.034 (2)	0.0143 (14)	−0.0059 (14)	0.0030 (12)	0.0001 (13)
C4	0.0238 (15)	0.0237 (19)	0.0217 (15)	0.0021 (13)	0.0026 (12)	0.0045 (13)
C5	0.0202 (14)	0.0213 (18)	0.0174 (14)	−0.0011 (12)	0.0042 (11)	0.0004 (12)
C6	0.0135 (12)	0.0231 (17)	0.0153 (13)	−0.0034 (11)	0.0038 (10)	−0.0015 (12)
C7	0.0118 (12)	0.0208 (17)	0.0188 (14)	−0.0013 (11)	0.0052 (10)	−0.0005 (12)
C8	0.0128 (12)	0.0182 (15)	0.0154 (13)	−0.0015 (11)	0.0054 (10)	−0.0001 (12)
C9	0.0159 (13)	0.0178 (16)	0.0159 (13)	−0.0009 (11)	0.0056 (10)	−0.0009 (12)
C10	0.0133 (13)	0.0220 (17)	0.0196 (14)	0.0027 (11)	0.0031 (10)	0.0004 (12)
C11	0.0187 (14)	0.0282 (19)	0.0151 (14)	0.0018 (12)	0.0008 (11)	0.0015 (12)
C12	0.0217 (15)	0.0285 (19)	0.0165 (14)	−0.0005 (13)	0.0062 (11)	0.0000 (13)
C13	0.0178 (13)	0.0252 (17)	0.0174 (14)	−0.0012 (12)	0.0078 (11)	−0.0002 (13)
C14	0.0147 (13)	0.0189 (16)	0.0167 (13)	−0.0017 (11)	0.0025 (10)	−0.0001 (12)
C15	0.0163 (13)	0.0233 (17)	0.0193 (14)	−0.0008 (12)	0.0052 (11)	−0.0024 (13)
C16	0.0244 (15)	0.0222 (17)	0.0179 (14)	−0.0001 (13)	0.0077 (11)	−0.0044 (13)
C17	0.0272 (15)	0.0188 (17)	0.0164 (14)	−0.0041 (13)	0.0028 (12)	−0.0037 (12)
C18	0.0169 (13)	0.0204 (17)	0.0226 (15)	−0.0024 (12)	0.0008 (11)	−0.0021 (13)
C19	0.0143 (13)	0.0194 (16)	0.0186 (14)	0.0016 (11)	0.0028 (10)	−0.0010 (12)
C20	0.0155 (12)	0.0156 (15)	0.0138 (13)	−0.0010 (11)	0.0038 (10)	0.0004 (11)
C21	0.0139 (12)	0.0137 (15)	0.0145 (13)	0.0017 (11)	0.0028 (10)	0.0004 (11)
C22	0.0117 (12)	0.0154 (15)	0.0130 (12)	0.0013 (11)	0.0037 (9)	0.0006 (11)
C23	0.0110 (12)	0.0169 (15)	0.0140 (13)	0.0014 (11)	0.0037 (10)	−0.0002 (11)
C24	0.0146 (13)	0.0199 (16)	0.0174 (14)	−0.0011 (11)	0.0025 (10)	−0.0034 (12)
C25	0.0168 (13)	0.0177 (16)	0.0176 (14)	−0.0020 (12)	0.0014 (10)	−0.0036 (12)
C26	0.0249 (15)	0.0189 (16)	0.0134 (13)	0.0010 (12)	0.0054 (11)	−0.0009 (12)
C27	0.0168 (13)	0.0188 (16)	0.0167 (13)	0.0003 (11)	0.0069 (10)	−0.0003 (12)
C28	0.0125 (12)	0.0165 (15)	0.0144 (13)	−0.0011 (11)	0.0020 (10)	0.0019 (11)
C29	0.0312 (17)	0.031 (2)	0.0234 (16)	0.0061 (15)	0.0072 (13)	0.0029 (15)
C30	0.0370 (19)	0.030 (2)	0.0317 (19)	0.0006 (16)	0.0035 (15)	−0.0049 (16)
C31	0.0349 (19)	0.039 (2)	0.036 (2)	−0.0051 (17)	0.0118 (15)	−0.0035 (18)

### Geometric parameters (Å, °)

**Table:**

Cu1—O2	1.918 (2)	C9—C14	1.402 (4)
Cu1—O1	1.944 (2)	C10—C11	1.384 (4)
Cu1—N5	1.945 (2)	C10—H10	0.9500
Cu1—N2	1.956 (2)	C11—C12	1.407 (4)
Cu1—O3	2.855 (3)	C11—H11	0.9500
O1—C7	1.281 (3)	C12—C13	1.391 (4)
O2—C21	1.280 (3)	C12—H12	0.9500
O3—C29	1.223 (4)	C13—C14	1.398 (4)
N1—C7	1.321 (4)	C13—H13	0.9500
N1—C8	1.365 (4)	C15—C16	1.389 (4)
N1—H1N	0.8800	C15—C20	1.403 (4)
N2—C8	1.341 (3)	C15—H15	0.9500
N2—C9	1.397 (3)	C16—C17	1.392 (4)
N3—C8	1.363 (4)	C16—H16	0.9500
N3—C14	1.388 (4)	C17—C18	1.383 (4)
N4—C21	1.325 (3)	C17—H17	0.9500
N4—C22	1.363 (4)	C18—C19	1.385 (4)
N4—H4N	0.8800	C18—H18	0.9500
N5—C22	1.357 (3)	C19—C20	1.396 (4)
N5—C23	1.398 (4)	C19—H19	0.9500
N6—C22	1.360 (3)	C20—C21	1.494 (4)
N6—C28	1.384 (4)	C23—C24	1.394 (4)
N7—C29	1.336 (4)	C23—C28	1.400 (4)
N7—C30	1.444 (5)	C24—C25	1.393 (4)
N7—C31	1.461 (4)	C24—H24	0.9500
C1—C2	1.385 (4)	C25—C26	1.397 (4)
C1—C6	1.404 (4)	C25—H25	0.9500
C1—H1	0.9500	C26—C27	1.384 (4)
C2—C3	1.385 (5)	C26—H26	0.9500
C2—H2	0.9500	C27—C28	1.393 (4)
C3—C4	1.395 (5)	C27—H27	0.9500
C3—H3	0.9500	C29—H29	0.9500
C4—C5	1.378 (4)	C30—H30A	0.9800
C4—H4	0.9500	C30—H30B	0.9800
C5—C6	1.400 (4)	C30—H30C	0.9800
C5—H5	0.9500	C31—H31A	0.9800
C6—C7	1.492 (4)	C31—H31B	0.9800
C9—C10	1.397 (4)	C31—H31C	0.9800
			
O2—Cu1—O1	162.00 (10)	C11—C12—H12	119.3
O2—Cu1—N5	89.19 (9)	C12—C13—C14	116.7 (3)
O1—Cu1—N5	93.79 (9)	C12—C13—H13	121.6
O2—Cu1—N2	92.89 (9)	C14—C13—H13	121.6
O1—Cu1—N2	89.46 (9)	N3—C14—C13	131.5 (3)
N5—Cu1—N2	162.91 (11)	N3—C14—C9	106.1 (2)
O3—Cu1—O1	84.70 (9)	C13—C14—C9	122.4 (3)
O3—Cu1—O2	77.75 (8)	C16—C15—C20	120.4 (3)
O3—Cu1—N2	84.97 (9)	C16—C15—H15	119.8
O3—Cu1—N5	112.03 (10)	C20—C15—H15	119.8
C7—O1—Cu1	126.5 (2)	C15—C16—C17	120.0 (3)
C21—O2—Cu1	130.33 (18)	C15—C16—H16	120.0
C7—N1—C8	120.2 (2)	C17—C16—H16	120.0
C7—N1—H1N	119.9	C18—C17—C16	119.9 (3)
C8—N1—H1N	119.9	C18—C17—H17	120.1
C8—N2—C9	105.7 (2)	C16—C17—H17	120.1
C8—N2—Cu1	122.08 (19)	C17—C18—C19	120.2 (3)
C9—N2—Cu1	131.98 (18)	C17—C18—H18	119.9
C8—N3—C14	107.3 (2)	C19—C18—H18	119.9
C21—N4—C22	119.5 (2)	C18—C19—C20	120.8 (3)
C21—N4—H4N	120.3	C18—C19—H19	119.6
C22—N4—H4N	120.3	C20—C19—H19	119.6
C22—N5—C23	105.7 (2)	C19—C20—C15	118.6 (3)
C22—N5—Cu1	123.32 (19)	C19—C20—C21	123.0 (3)
C23—N5—Cu1	130.99 (18)	C15—C20—C21	118.5 (2)
C22—N6—C28	108.0 (2)	O2—C21—N4	127.3 (3)
C29—N7—C30	123.3 (3)	O2—C21—C20	114.7 (2)
C29—N7—C31	120.3 (3)	N4—C21—C20	118.0 (2)
C30—N7—C31	116.3 (3)	N5—C22—N6	111.1 (2)
C2—C1—C6	120.2 (3)	N5—C22—N4	130.0 (2)
C2—C1—H1	119.9	N6—C22—N4	118.8 (2)
C6—C1—H1	119.9	C24—C23—N5	130.9 (2)
C3—C2—C1	120.4 (3)	C24—C23—C28	120.1 (3)
C3—C2—H2	119.8	N5—C23—C28	109.0 (2)
C1—C2—H2	119.8	C25—C24—C23	118.2 (3)
C2—C3—C4	119.4 (3)	C25—C24—H24	120.9
C2—C3—H3	120.3	C23—C24—H24	120.9
C4—C3—H3	120.3	C24—C25—C26	120.5 (3)
C5—C4—C3	120.8 (3)	C24—C25—H25	119.7
C5—C4—H4	119.6	C26—C25—H25	119.7
C3—C4—H4	119.6	C27—C26—C25	122.1 (3)
C4—C5—C6	120.0 (3)	C27—C26—H26	118.9
C4—C5—H5	120.0	C25—C26—H26	118.9
C6—C5—H5	120.0	C26—C27—C28	116.7 (3)
C5—C6—C1	119.1 (3)	C26—C27—H27	121.6
C5—C6—C7	120.4 (3)	C28—C27—H27	121.6
C1—C6—C7	120.5 (3)	N6—C28—C27	131.6 (2)
O1—C7—N1	127.9 (3)	N6—C28—C23	106.2 (2)
O1—C7—C6	116.3 (3)	C27—C28—C23	122.2 (3)
N1—C7—C6	115.7 (2)	O3—C29—N7	127.4 (3)
N2—C8—N3	111.9 (2)	O3—C29—H29	116.3
N2—C8—N1	129.8 (3)	N7—C29—H29	116.3
N3—C8—N1	118.3 (2)	N7—C30—H30A	109.5
C10—C9—N2	131.1 (3)	N7—C30—H30B	109.5
C10—C9—C14	119.9 (3)	H30A—C30—H30B	109.5
N2—C9—C14	109.0 (2)	N7—C30—H30C	109.5
C11—C10—C9	118.3 (3)	H30A—C30—H30C	109.5
C11—C10—H10	120.9	H30B—C30—H30C	109.5
C9—C10—H10	120.9	N7—C31—H31A	109.5
C10—C11—C12	121.2 (3)	N7—C31—H31B	109.5
C10—C11—H11	119.4	H31A—C31—H31B	109.5
C12—C11—H11	119.4	N7—C31—H31C	109.5
C13—C12—C11	121.4 (3)	H31A—C31—H31C	109.5
C13—C12—H12	119.3	H31B—C31—H31C	109.5

### Hydrogen-bond geometry (Å, °)

**Table:**

*D*—H···*A*	*D*—H	H···*A*	*D*···*A*	*D*—H···*A*
N1—H1N···N6^i^	0.88	2.05	2.847 (3)	151
N4—H4N···N3^ii^	0.88	2.26	3.019 (3)	144

Symmetry codes: (i) *x*−1, *y*, *z*; (ii) *x*+1, *y*, *z*.
